# Relationship Between Tumor Necrosis Factor-α rs361525 Polymorphism and Gastric Cancer Risk: A Meta-Analysis

**DOI:** 10.3389/fphys.2018.00469

**Published:** 2018-05-15

**Authors:** Tianshu Xu, Zhijun Kong, Hui Zhao

**Affiliations:** ^1^Department of Traditional Chinese Medicine, Nanjing Drum Tower Hospital, The Affiliated Hospital of Nanjing University Medical School, Nanjing, China; ^2^Department of General Surgery, Affiliated Hospital of Nanjing Medical University, Changzhou Second People's Hospital, Changzhou, China; ^3^Department of General Surgery, Wuxi Third People's Hospital, Wuxi, China

**Keywords:** tumor necrosis factor α, gene polymorphism, meta-analysis, gastric cancer, false-positive report probability

## Abstract

Tumor necrosis factor (TNF)-α, a major part in inflammatory, infectious and tumor processes, and is pivotal at the early stages of gastric cancer. Relationship between its risk and *TNF-*α rs361525 polymorphism has been demonstrated, but remains conflicting and controversial. Therefore, a meta-analysis was conducted to more precisely estimate this relationship. PubMed, Web of Science, EMBASE and CNKI were comprehensively searched to find out relevant articles through October 5, 2017. The strength of the relationship was assessed using pooled odds ratios and 95% confidence intervals. Totally 20 articles were included involving 4,084 cases and 7,010 controls. No significant relationship between *TNF-*α rs361525 polymorphism and increased GC risk was found in the whole populations. Subgroup analyses uncovered *TNF-*α rs361525 polymorphism intensified the risk of GC among Asians under five models, but decreased the risk of GC among Caucasiansin the allelic and dominant models. Subgroup analysis by genotyping methods revealed increased risk for other methods. In conclusion, this meta-analysis suggests *TNF-*α rs361525 polymorphism is related to the risk of GC, especially for Asians.

## Introduction

Gastric cancer (GC) is the fourth major malignancy and the second dominant cause of cancer-induced death in the world (de Martel et al., 2012). In 2017, 28,000 new cases and 10,960 deaths of GC were projected to occur in the United States (Siegel et al., 2017). *Helicobacter pylori* infection contributes to causing the progression of chronic inflammation to GC (Fox and Wang, 2007). Some studies demonstrated the extremely low risk of developing GC in *H. pylori*- negative subjects (Uemura et al., 2002). However, nearly all *H. pylori*-positive subjects have chronic gastritis, and only 1–2% develop to GC. Therefore, other factors such as genetic factors and lifestyle may play important roles in the gastric tumorigenesis (Carcas, 2014).

Tumor necrosis factor (TNF)-α belonging to the TNF/TNF receptor cytokine superfamily can be found in plasma or serum of healthy people, as well as some cancer patients (Balkwill, 2006). TNF-α production by tumors is related with hormone irresponsiveness, poor prognosis, and cachexia/asthenia (Szlosarek and Balkwill, 2003; Tisdale, 2004). The TNF-α-blocked experimental mice were resistant to skin and colorectal cancer occurrence (Moore et al., 1999; Popivanova et al., 2008). (Oshima et al., 2014). The TNF-α/TNFR1 signaling could promote GC occurrence by inducing NADPH oxidase organizer 1 (Noxo1) and G protein subunit alpha 14 (GNA14), which are crucial in the tumorigenicity and stemness of GC cells, in tumor cells (Oshima et al., 2014). The above observations suggest TNF-α plays important roles in the etiology of GC.

*TNF-*α located in chromosome 2 has four exon counts. The gene contains several polymorphic sites, including the widely-studied *TNF-*α-238 (rs361525) and−308 (rs1800629). Some studies demonstrated the relationship between *TNF-*α rs361525 polymorphism and GC risk (Zambon et al., 2005; Xing et al., 2006; Zeng et al., 2007; Bai et al., 2009; Yin et al., 2012), but other studies have found no such relationship (Jang et al., 2001; Wu et al., 2002, 2003, 2004; Glas et al., 2004; Lee et al., 2004; Lu et al., 2005; Kamangar et al., 2006; Garcia-Gonzalez et al., 2007; Hou et al., 2007; Crusius et al., 2008; Yang et al., 2009; Whiteman et al., 2010; Essadik et al., 2015; Xu et al., 2017). This inconsistency may be attributed to weak statistical power, small sample size, and clinical heterogeneity. Therefore, a meta-analysis was conducted to overcome the limitations of individual studies and clarify whether *TNF-*α rs361525 polymorphism conferred susceptibility to GC.

## Materials and methods

### Literature search

Two investigators systematically searched PubMed, Elsevier, EMBASE, and CNKI through Oct 5, 2017 to identify relevant studies using the following search terms: “Gastric Neoplasm,” “Stomach Cancer,” “Gastric Cancer,” “Gastric Carcinoma,” “Gastric Adenocarcinoma,” “tumor necrosis factor alpha,” “*TNF-*α,” “polymorphism,” “SNP,” and “variant.” In addition, all cited references were reviewed to find out studies that were not included in the above electronic databases. When two studies overlapped, we chose the latest study or the one with larger sample size. There was no restriction on language, ethnicity or region of study population. GC was diagnosed according to classification criteria.

### Inclusion and exclusion criteria

Inclusion criteria were: (1) evaluation of relationship between GC risk and TNF-α rs361525 polymorphism; (2) study on humans; (3) provision of enough data for computation of odds ratios (ORs) and 95% confidence intervals (CIs); (4) case- control study. Exclusion criteria were: (1) duplication; (2) case report or review; (3) lack of genotype data; (4) irrelevant topic.

### Data extraction and quality assessment

From each included study, data including name of first author, country of origin, publication year, ethnicity, age, and genotype numbers in cases and controls was extracted. When more than one ethnicity were involved, genotype data was processed separately. Data extraction and study quality assessment based on the Newcastle-Ottawa Scale (NOS) (Stang, 2010) were conducted by two investigators independently. The NOS score varies from 0 up to 9: high-quality study: >7; medium-quality study: 4–6; poor-quality study: <4. All conflicting information was discussed and resolved with consensus.

### Genotype and gene expression correlation analysis

Genotype data of TNF-α rs361525 polymorphism and its mRNA expression data were available from the International HapMap Project and GTex portal (https://www.gtexportal.org/home/), respectively (Gong et al., 2017).

### Statistical analysis

The strength of relationship between GC risk and *TNF-*α rs361525 polymorphism was investigated by using crude ORs and 95%CIs. The following comparisons for this relationship were made: the dominant model (AA+GA vs. GG), the recessive model (AA vs. GA+GG), the heterozygote model (GA vs. GG), the homozygote model (AA vs. GG), and the allele model (A vs. G). Stratification analyses were carried out by ethnicity, source of control (SOC), Hardy-Weinberg equilibrium (HWE), genotyping method and NOS score (He et al., 2014). The null hypothesis that all studies evaluated the same effect was tested by Cochran's Q-statistics. When significant heterogeneity was found (*P* > 0.10 or I^2^ > 50%), a random-effect model was used; otherwise, a fixed-effect model was applied (Higgins and Thompson, 2002). Sensitivity analysis was conducted by omitting one study at a time to test the relative influence on the pooled estimate. The significant findings were evaluated by calculating false-positive report probability (FPRP). An FPRP threshold of 0.2 and a prior probability of 0.1 were set to detect an OR for a correlation with the tested genotype. FPRP <0.2 implied a significant relationship (He et al., 2013). χ^2^ test was carried out to clarify whether the observed genotype frequencies conformed to the HWE. Potential publication bias was examined by Begger's and Egger's linear regression tests (Wassen and Jertborn, 2006), with the significant level at *P* < 0.05. All statistical analyses were conducted on Stata 11.0 (StataCorp, College Station, USA).

## Results

### Study characteristics

**Figure 1:**
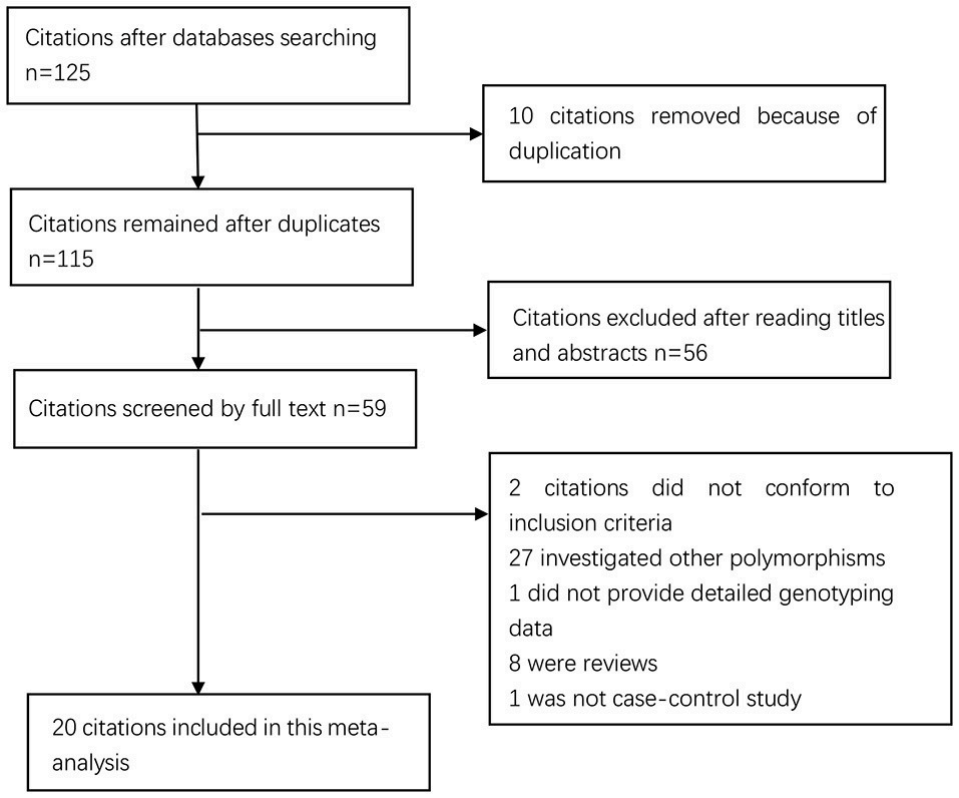
Selection for eligible papers included in this meta-analysis.

The process of study selection is shown in Figure 1. The initial search returned 125 studies. Of them, 10 duplicates and 56 papers unrelated to the topic based on their abstracts and titles were excluded. Among the remaining 59 papers: 2 papers did not meet inclusion criteria; 27 papers investigated other polymorphisms; 1 paper 1 paper was not case-control study; 1 paper did not provide enough data. Finally, 20 4,084 cases and 7,010 controls were included. Of them, 12 articles were from Asian populations (Jang et al., 2001; Wu et al., 2002, 2003, 2004; Lee et al., 2004; Lu et al., 2005; Xing et al., 2006; Zeng et al., 2007; Bai et al., 2009; Yang et al., 2009; Yin et al., 2012; Xu et al., 2017) and 8 from Caucasian populations (Glas et al., 2004; Zambon et al., 2005; Kamangar et al., 2006; Garcia-Gonzalez et al., 2007; Hou et al., 2007; Crusius et al., 2008; Whiteman et al., 2010; Essadik et al., 2015). Six studies failed to obey HWE (Wu et al., 2002, 2003, 2004; Kamangar et al., 2006; Garcia-Gonzalez et al., 2007; Whiteman et al., 2010). The details of the included studies are presented in Table 1. The year of publication ranged from 2001 to 2012. The numbers of cases and controls ranged from 52 to 404 and from 74 to 1,299, respectively.

**Table 1:** Characteristics of included studies.

**Author and year**	**Gender (Female/Male)**		**Age**		**SOC**	**Nationality**	**Ethnicity**	**Case/Control**	**Genotyping method**	**HWE**	**NOS**
	**Case**	**Control**	**Case**	**Control**							
Jang et al., 2001	N/A	N/A	N/A	N/A	HB	Korea	Asian	52/92	PCR-RFLP	0.391	6
Wu et al., 2002	N/A	N/A	N/A	N/A	HB	Taiwan	Asian	150/220	Sequencing	<0.001	5
Wu et al., 2003	84/136	88/142	60.9 ± 12.6	60.7 ± 13.4	HB	Taiwan	Asian	220/230	Sequencing	<0.001	6
Glas et al., 2004	71/74	41/47	65 ± 12.5	45 ± 12.5	HB	Germany	Caucasian	145/88	PCR-RFLP	0.635	6
Lee et al., 2004	142/199	123/138	46.0 ± 12.6	48.7 ± 10.9	HB	Korea	Asian	341/261	Sequencing	0.416	6
Wu et al., 2004	78/126	84/126	60.1 ± 12.1	58.7 ± 14.4	HB	Taiwan	Asian	204/210	Sequencing	<0.001	6
Lu et al., 2005	67/183	83/217	59.0 ± 12.3	59.1 ± 9.4	PB	China	Asian	250/300	DHPLC	0.49	7
Zambon et al., 2005	N/A	N/A	N/A	N/A	HB	Italy	Caucasian	129/644	TaqMan	0.378	6
Kamangar et al., 2006	N/A	N/A	N/A	N/A	PB	Finland	Caucasian	210/115	TaqMan	<0.001	7
Zambon et al., 2005	60/70	72/70	58.6 ± 13.3	53.5 ± 11.2	HB	China	Asian	130/142	gene chip	0.23	6
Hou et al., 2007	103/202	152/275	<50 39 50–59 56 60–69 120 ≥70 90	<50 52 50–59 75 60–69 168 ≥70 132	PB	Poland	Caucasian	299/412	TaqMan	0.492	6
Garcia-Gonzalez et al., 2007	146/258	138/266	73.7 ± 10.3	71.3 ± 12.0	HB	Spain	Caucasian	404/404	TaqMan	0.011	6
Zeng et al., 2007	60/70	72/70	59.0 ± 13.0	54.0 ± 11.0	HB	China	Asian	130/142	gene chip	0.23	7
Crusius2008	N/A	N/A	N/A	N/A	PB	Europe	Caucasian	235/1123	TaqMan	0.367	8
Yang et al., 2009	25/59	100/236	≤ 63 43 >63 41	≤63 176 >63 160	PB	Korea	Asian	83/331	SNaPshot	0.457	6
Bai et al., 2009	50/64	56/63	58.3 ± 12.5	55.9 ± 14.9	HB	China	Asian	114/119	gene chip	0.668	6
Whiteman et al., 2010	22/247	459/896	<49 21 50–59 75 60–69 103 70–79 70	<49 216 50–59 348 60–69 480 70–79 311	PB	Australia	Caucasian	289/1299	gene chip	0.007	7
Yin et al., 2012	N/A	N/A	N/A	N/A	HB	China	Asian	310/485	SNaPshot	0.369	6
Essadik et al., 2015	N/A	N/A	N/A	N/A	PB	Morocco	Caucasian	93/74	Sequencing	0.978	7
Xu et al., 2017	169/127	180/139	44.0 ± 16.6	44.3 ± 15.9	HB	China	Asian	294/319	PCR-RFLP	0.466	6

*SOC, source of controls; PB, population-based controls; HB, hospital-based controls; NOS, Newcastle-Ottawa Scale; HWE, Hardy–Weinberg equilibrium; PCR-RFLR, PCR-restriction fragment length polymorphism; DHPLC, Denaturing high-performance liquid chromatograph*.

### Quantitative analysis

**Figure 2:**
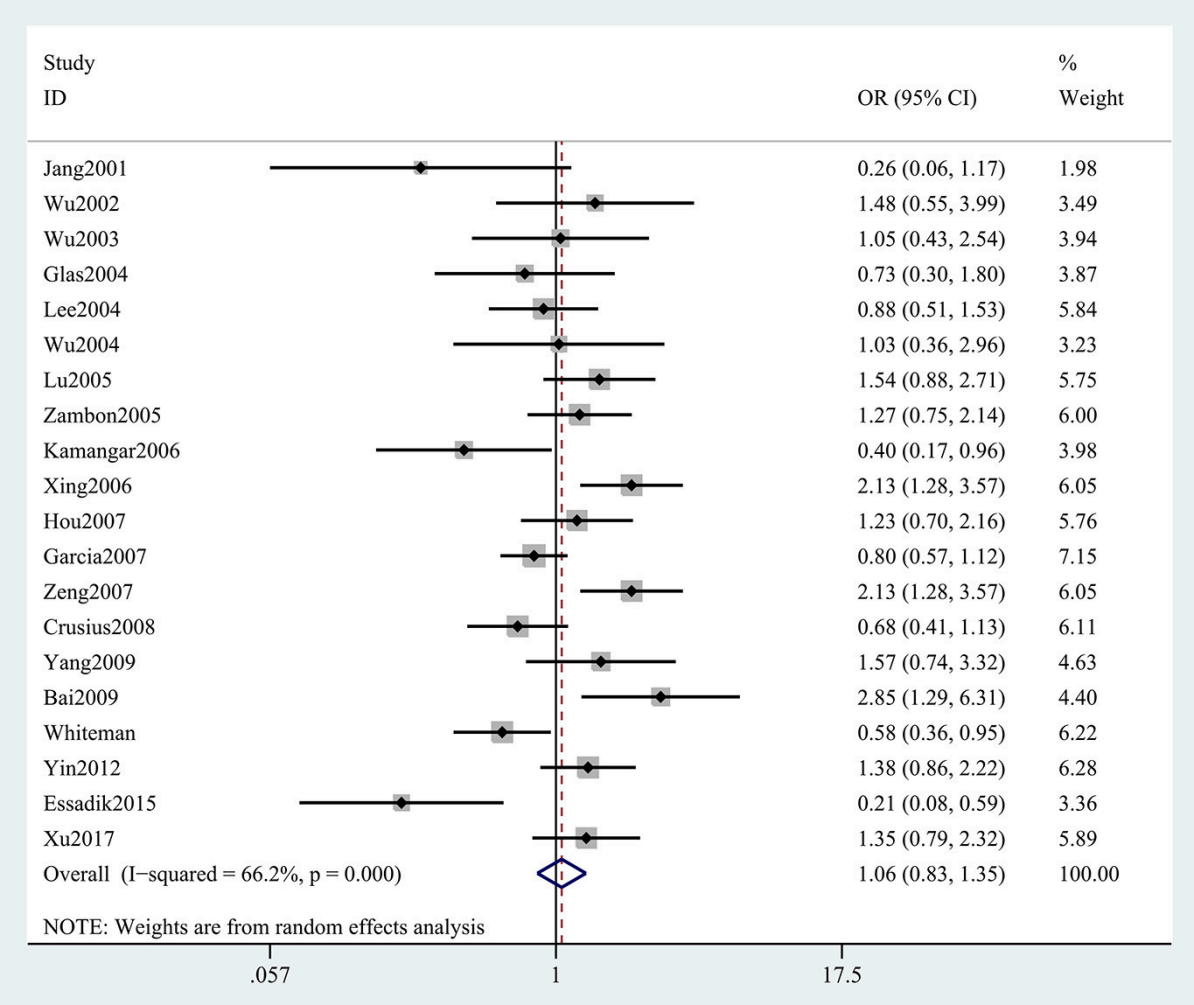
Forest plot shows odds ratio for the association between TNF-α rs361525 polymorphism and GC risk (A vs. G).

**Figure 3:**
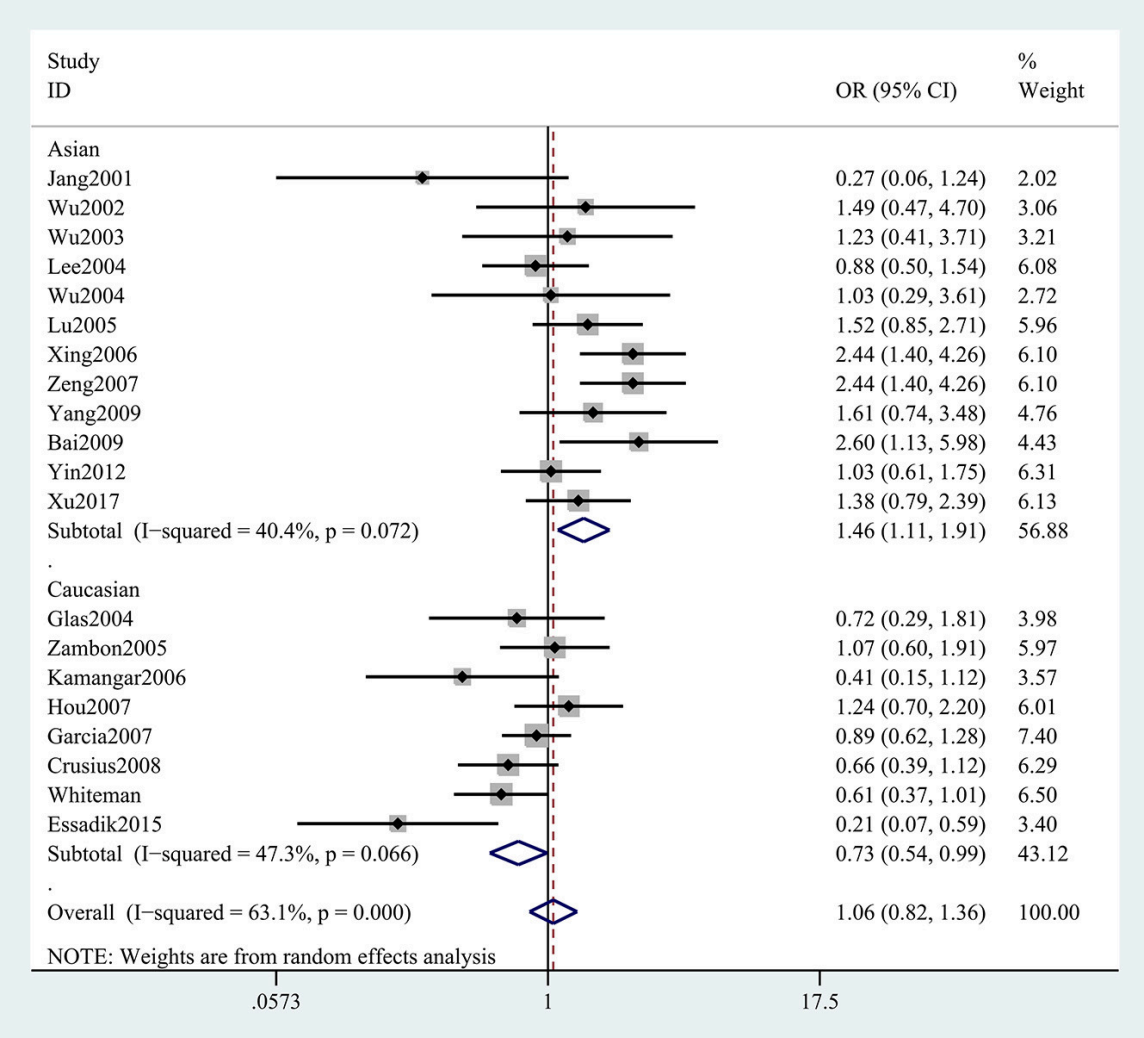
Stratification analyses of ethnicity between TNF-α rs361525 polymorphism and GC risk (AA+GA vs. GG).

**Figure 4:**
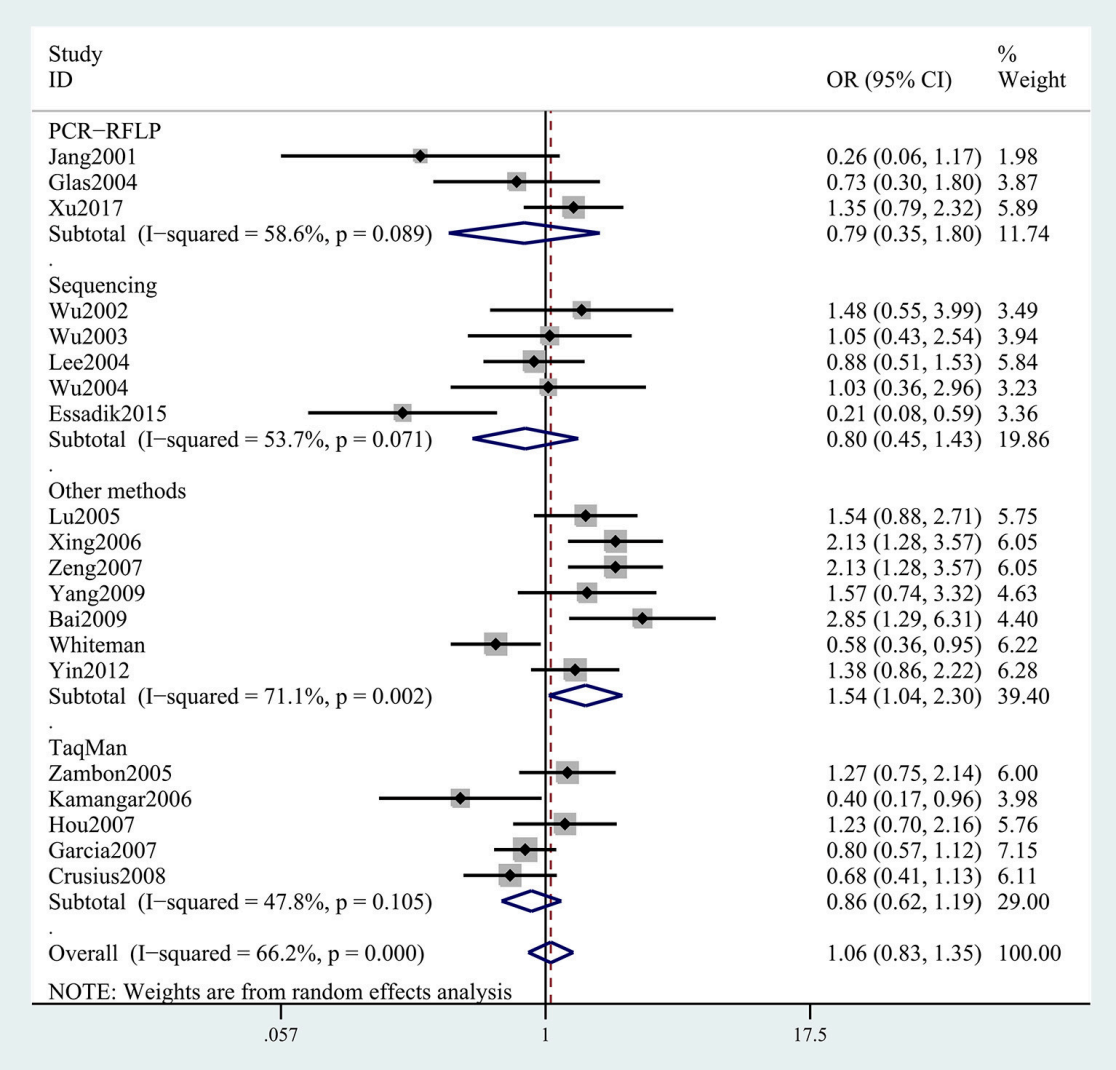
Stratification analyses of genotyping methods between TNF-α rs361525 polymorphism and GC risk (A vs. G).

The results concerning the relationship between *TNF-*α rs361525 polymorphism and GC risk were summarized in Tables 2, 3. This relationship was insignificant in the overall population (A vs. G: OR, 1.06; 95%CI, 0.83–1.35, *P* = 0.646, Figure 2). Stratification analyses of ethnicity indicated *TNF-*α rs361525 polymorphism intensified the risk of GC among Asians in most of the comparisons (AA+GA vs. GG: OR, 1.46; 95%CI, 1.11–1.91, *P* = 0.007, Figure 3), but decreased the risk among Caucasians in the allele and dominant models (AA+GA vs. GG: OR, 0.73; 95% CI, 0.54–0.99, *P* = 0.043, Table 3). Subgroup analyses by genotyping methods revealed increased risk for other methods (A vs. G: OR, 1.54; 95%CI, 1.04–2.30, *P* = 0.033, Figure 4) and this relationship also held true in the HWE-positive studies. Stratified analysis by SOC did not find significant correlation in the hospital- or population-based studies (Table 3). Similar results were observed in the subgroup analysis of NOS score.

**Table 2:** Meta-analysis of association between TNF-α rs361525 polymorphism and gastric cancer.

**Comparison**	**OR(95%CI)**	***P*-value**	***^a^P*-value**	***P* for heterogeneity**	**I^2^ (%)**	**Model**
A vs. G	1.06(0.83,1.35)	0.646	1.000	<0.001	66.2	Random
AA+GA vs. GG	1.06(0.83,1.36)	0.657	1.000	<0.001	63.1	Random
AA vs. GA+GG	1.14(0.70,1.85)	0.782	0.782	0.053	42.4	Fixed
AA vs. GG	1.12(0.69,1.83)	0.644	1.000	0.047	43.5	Fixed
GA vs. GG	1.05(0.81,1.34)	0.733	0.917	<0.001	60.2	Random

a *P values were calculated by a multiple comparison of Bonferroni correction*.

**Table 3:** Summary of the subgroup analyses in this meta-analysis.

**Comparisons**	**Category**	**Category**	**Studies**	**OR (95% CI)**	***P*-value**	***P* for heterogeneity**	**I^2^(%)**
A vs. G	Ethnicity	Asian	12	**1.46(1.16, 1.85)**	0.002	0.054	32.8
		Caucasian	8	**0.72(0.53, 0.99)**	0.043	0.106	56.4
	SOC	HB	13	1.26(0.97, 1.64)	0.084	0.117	55.3
		PB	7	0.77(0.49, 1.22)	0.271	0.265	77.2
	HWE	Positive	14	1.19(0.90, 1.59)	0.226	0.183	65.7
		Negative	6	0.76(0.57, 1.00)	0.051	0.019	14.8
	Genotyping	PCR-RFLR	3	0.80(0.35, 1.80)	0.582	0.299	58.6
		Sequencing	5	0.80(0.45, 1.43)	0.455	0.223	53.7
		TagMan	5	0.86(0.62, 1.19)	0.355	0.063	47.8
		Other methods	7	**1.54(1.04, 2.30)**	0.033	0.200	71.1
	NOS score	5 ≤ Score ≤ 6	14	1.22(0.97, 1.53)	0.054	0.047	42.5
		Score > 6	6	0.75(0.41, 1.37)	0.185	<0.001	83.2
GA+AA vs. GG	Ethnicity	Asian	12	**1.46(1.11, 1.91)**	0.007	0.086	40.4
		Caucasian	8	**0.73(0.54, 0.99)**	0.043	0.082	47.3
	SOC	HB	13	1.25(0.95, 1.65)	0.111	0.122	52.6
		PB	7	0.79(0.50, 1.24)	0.301	0.253	69.6
	HWE	Positive	14	1.16(0.85, 1.57)	0.357	0.216	67.0
		Negative	6	0.80(0.62, 1.04)	0.095	<0.001	0.0
	Genotyping	PCR-RFLR	3	0.81(0.36, 1.83)	0.606	0.291	56.8
		Sequencing	5	0.80(0.42, 1.50)	0.479	0.262	51.8
		TagMan	5	0.87(0.66, 1.15)	0.333	0.025	24.1
		Other methods	7	1.53(0.99, 2.36)	0.054	0.243	72.6
	NOS score	5 ≤ Score ≤ 6	14	1.20(0.95, 1.50)	0.120	0.105	33.8
		Score >6	6	0.77(0.41, 1.45)	0.414	<0.001	82.8
AA vs. GA+GG	Ethnicity	Asian	7	**2.41(1.16, 4.98)**	0.018	0.300	17.1
		Caucasian	6	0.51(0.23, 1.12)	0.095	0.040	57.2
	SOC	HB	8	1.50(0.84, 2.67)	0.172	0.021	57.5
		PB	5	0.53(0.19, 1.48)	0.226	0.673	0.0
	HWE	Positive	7	**3.82(1.69, 8.61)**	0.001	0.172	33.6
		Negative	6	**0.45(0.21, 0.93)**	0.031	0.457	0.0
	Genotyping	PCR-RFLR	1	0.58(0.02, 14.52)	0.741	N/A	N/A
		Sequencing	4	0.86(0.33, 2.25)	0.761	0.835	0.0
		TagMan	4	0.61(0.26, 1.42)	0.247	0.012	72.5
		Other methods	4	**3.43(1.37, 8.61)**	0.009	0.127	47.4
	NOS score	5 ≤ Score ≤ 6	8	1.50(0.84, 2.67)	0.172	0.021	57.5
		Score>6	5	0.53(0.19, 1.48)	0.226	0.673	0.0
AA vs. GG	Ethnicity	Asian	7	**2.41(1.17, 4.98)**	0.018	0.291	18.3
		Caucasian	6	0.50(0.23, 1.10)	0.084	0.039	57.4
	SOC	HB	8	1.50(0.84, 2.67)	0.171	0.021	57.6
		PB	5	0.51(0.18, 1.43)	0.199	0.641	0.0
	HWE	Positive	7	**3.68(1.64, 8.28)**	0.002	0.146	37.0
		Negative	6	**0.44(0.21, 0.92)**	0.029	0.446	0.0
	Genotyping	PCR-RFLR	1	0.53(0.02, 13.30)	0.700	N/A	N/A
		Sequencing	4	0.85(0.33, 2.21)	0.740	0.794	0.0
		TagMan	4	0.60(0.26, 1.40)	0.236	0.013	72.4
		Other methods	4	**3.40(1.36, 8.47)**	0.009	0.121	48.4
	NOS score	5 ≤ Score ≤ 6	8	1.50(0.84, 2.67)	0.171	0.021	57.6
		Score>6	5	0.51(0.18, 1.43)	0.199	0.641	0.0
GA vs. GG	Ethnicity	Asian	12	**1.40(1.03, 1.91)**	0.032	0.032	48.0
		Caucasian	8	0.76(0.57, 1.01)	0.057	0.115	39.6
	SOC	HB	13	1.21(0.90, 1.63)	0.210	0.011	53.7
		PB	7	0.82(0.53, 1.26)	0.358	0.009	64.9
	HWE	Positive	14	1.09(0.80, 1.50)	0.585	<0.001	67.6
		Negative	6	0.87(0.66, 1.15)	0.322	0.466	0.0
	Genotyping	PCR-RFLR	3	0.84(0.38, 1.82)	0.651	0.651	52.6
		Sequencing	5	0.80(0.39, 1.66)	0.553	0.553	49.0
		TagMan	5	0.90(0.69, 1.16)	0.411	0.411	7.6
		Other methods	7	1.43(0.91, 2.26)	0.120	0.120	73.9
	NOS score	5 ≤ Score ≤ 6	14	1.16(0.91, 1.47)	0.233	0.096	34.9
		Score>6	6	0.80(0.43, 1.48)	0.473	<0.001	80.8

*SOC, source of controls; PB, population-based controls; HB, hospital-based controls; NOS, Newcastle-Ottawa Scale; HWE, Hardy–Weinberg equilibrium; PCR-RFLR, PCR-restriction fragment length polymorphism. Bold values are statistically significant (P < 0.05)*.

### TNF-α mRNA expression by genotypes

**Figure 5:**
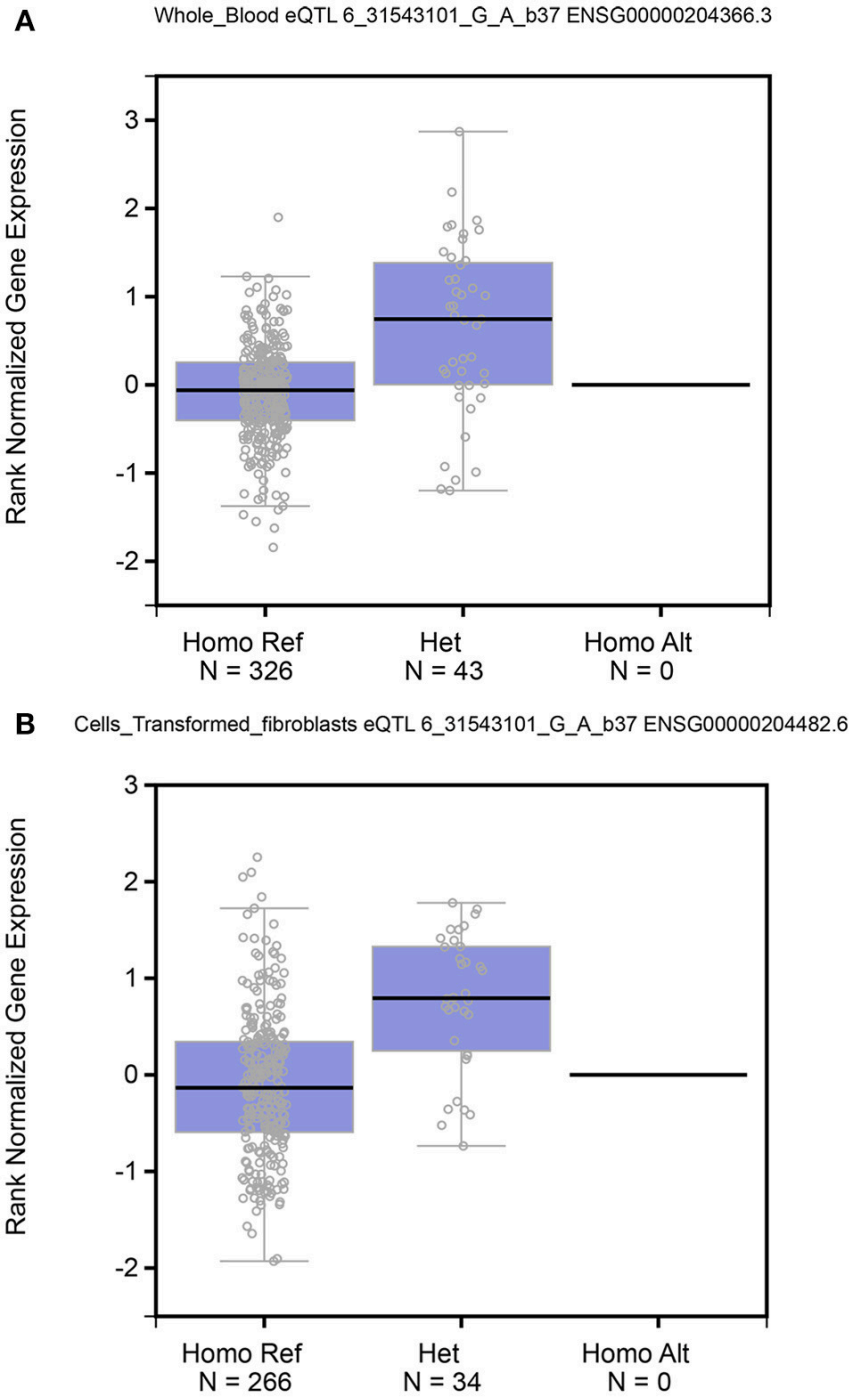
The TNF-α mRNA expression levels by the genotypes of rs361525 polymorphism.

The TNF-α mRNA expression levels by the genotypes of rs361525 polymorphism were significantly different for the whole blood (*P* = 5.10 × 10^−14^) and transformed fibroblasts (*P* = 6.65 × 10^−10^) (Figure 5).

### Sensitivity and publication bias

**Figure 6:**
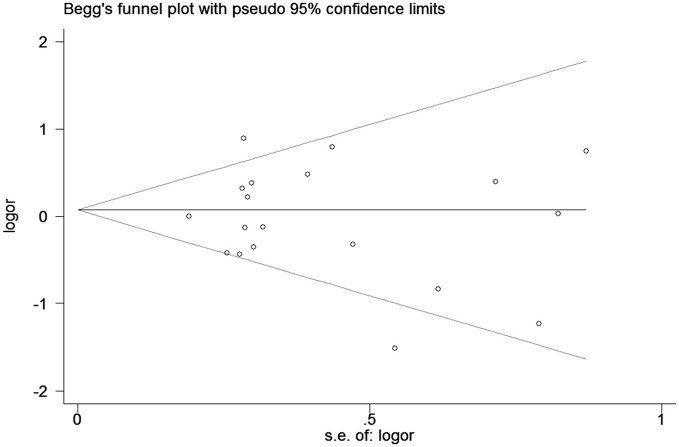
Begg's tests for publication bias between TNF-α rs361525 polymorphism and risk of GC (GA vs. GG).

In the sensitivity analysis, no overall significant change was found when any single study was removed, suggesting our results are statistically robust. Neither Egger's nor Begg's tests (GA vs. GG, Figure 6) showed any evidence of publication bias in this meta-analysis.

### FPRP analyses

The FPRPs for significant results at different *p* levels are shown in Table 4. At the level of 0.1, some FPRPs were all <0.20, indicating the significant associations between TNF-α rs361525 polymorphism and GC risk were noteworthy (Table 4). However, the FPRPs for other significant associations were larger, suggesting some possible bias due to sample size reduction existed in some subgroups, which should be validated by larger-size studies in the future.

**Table 4:** False-positive report probability values for associations between the TNF-α−238 polymorphism and gastric cancer risk.

**Variables**	**OR(95%CI)**	***P* value**	**Power**	**Prior Probability**				
				**0.25**	**0.1**	**0.01**	**0.001**	**0.0001**
**A vs. G**								
Asian	1.46(1.16, 1.85)	0.002	0.605	**0.010**	**0.029**	0.247	0.768	0.971
Caucasian	0.72(0.53, 0.99)	0.043	0.670	**0.161**	0.366	0.864	0.985	0.998
Other methods	1.54(1.04, 2.30)	0.033	0.565	**0.149**	0.344	0.852	0.983	0.998
**GA**+**AA vs. GG**								
Asian	1.46(1.11, 1.91)	0.007	0.603	**0.034**	**0.095**	0.535	0.921	0.991
Caucasian	0.73(0.54, 0.99)	0.043	0.710	**0.154**	0.353	0.857	0.984	0.998
**AA vs. GA**+**GG**								
Asian	2.41(1.16, 4.98)	0.018	0.543	**0.090**	0.230	0.766	0.971	0.997
HWE-positive	3.82(1.69, 8.61)	0.001	0.497	**0.006**	**0.018**	0.166	0.668	0.953
HWE-negative	0.45(0.21, 0.93)	0.031	0.624	**0.130**	0.309	0.831	0.980	0.998
Other methods	3.43(1.37, 8.61)	0.009	0.522	**0.049**	0.134	0.630	0.945	0.994
**AA vs. GG**								
Asian	2.41(1.17, 4.98)	0.018	0.543	**0.090**	0.230	0.766	0.971	0.997
HWE-positive	3.68(1.64, 8.28)	0.002	0.529	**0.011**	**0.033**	0.272	0.791	0.974
HWE-negative	0.44(0.21, 0.92)	0.029	0.628	**0.122**	0.293	0.820	0.979	0.998
Other methods	3.40(1.36, 8.47)	0.009	0.531	**0.048**	**0.132**	0.627	0.944	0.994
**GA vs. GG**								
Asian	1.40(1.03, 1.91)	0.032	0.661	**0.127**	0.304	0.827	0.980	0.988

*HWE, Hardy–Weinberg equilibrium*.

## Discussion

It is hypothesized chronic inflammation plays a crucial role in the etiology of GC and other cancers. Epplein et al. found the upregulated circulating levels of inflammation-related cytokines such as TNF-α may intensify the risk of GC (Epplein et al., 2013). TNF-α-induced protein secretion from *H. pylori* is involved in the development of GC (Suganuma et al., 2012). It is assumed the stimulation of the TNF-α/TNFR1 signaling in the tumor microenvironment enhances GC progression by inducing Noxo1 and GNA14 (Oshima et al., 2014). TNF-α is a promising effective target for GC therapy. The *TNF-*α rs361525 polymorphism could change TNF-α gene transcription and adjust TNF-α generation (Kaluza et al., 2000).

Many studies have reported the relationship between *TNF-*α rs361525 polymorphism and GC risk (Jang et al., 2001; Wu et al., 2002, 2003, 2004; Glas et al., 2004; Lee et al., 2004; Lu et al., 2005; Zambon et al., 2005; Kamangar et al., 2006; Xing et al., 2006; Garcia-Gonzalez et al., 2007; Hou et al., 2007; Zeng et al., 2007; Crusius et al., 2008; Bai et al., 2009; Yang et al., 2009; Whiteman et al., 2010; Yin et al., 2012; Essadik et al., 2015; Xu et al., 2017), but with conflicting findings. Given such conflicts, several meta-analyses in this field have been conducted (Zhou et al., 2011; Yu et al., 2013; Rokkas et al., 2014; Hui et al., 2016). Zhou et al. firstly meta-analyzed the association between *TNF-*α rs361525 polymorphism and cancer risk (Zhou et al., 2011), but found no significant result in the general populations (Zhou et al., 2011) or in the subgroups of cancer type including GC (Zhou et al., 2011). The two subsequent meta-analyses also did not observe an association between *TNF-*α rs361525 polymorphism and GC risk (Rokkas et al., 2014; Hui et al., 2016). Noticeably, Hui et al. revealed *TNF-*α rs361525 polymorphism could significantly increase the risk of digestive system cancer, but not GC (Hui et al., 2016). However, Yu et al. uncovered a significant relationship between *TNF-*α rs361525 polymorphism and increased GC risk in Asians, but not in Caucasians (Yu et al., 2013). Recently, new studies in this field have emerged, which further necessitates a new comprehensive meta-analysis. Here our data are consistent with Yu et al. It is worth noting that we found a significant association of *TNF-*α rs361525 polymorphism with GC among both Asians and Caucasians in the stratified analysis by ethnicity, while Yu et al. did not find any association among Caucasians (Yu et al., 2013). We found *TNF-*α rs361525 polymorphism increased the risk of GC among Asians, while this SNP seemed to protect Caucasians from GC. There are several possible interpretations for the different findings between Asians and Caucasians. Firstly, GC may be genetically and clinically heterogeneous among different populations. Secondly, varying sample sizes of Asians and Caucasians may also account. The third reason may be the differences in genotyping methods and random errors. The fourth reason may be the varying prevalence of *H. pylori* among different populations. Last but not least, different geographical environments and dietary pattern may also be influential (such as Asians eat more pickled and fried food). We believe this meta-analysis has several strengths over previous meta-analyses. Firstly, our sample size was larger. Secondly, sensitivity analysis proved the reliability and stability of our data. Thirdly, we conducted subgroup analyses of ethnicity, HWE, SOC, NOS score, and genotyping methods, and explored the potential sources of heterogeneity. Finally, we calculated false-positive report probability and statistical power.

However, this study also has some limitations. Firstly, no subgroup analyses of confounding factors such as age, sex, smoking or *H. pylori* infection were conducted. Secondly, potential gene-gene or gene-environment interactions were not assessed. Thirdly, only one SNP of *TNF-*α gene was investigated. Finally, the relationship between this SNP and clinical manifestations of GC was not examined.

In conclusion, this meta-analysis indicates the *TNF-*α rs361525 polymorphism increases the risk of GC among Asians, but decreases the risk of GC among Caucasians. This finding should be validated by larger case-control studies in other ethnicities.

## Author contributions

ZK conceived the entire study. TX and HZ analyzed the data. TX and ZK performed statistical analysis. ZK and HZ wrote the paper. All authors read and agreed with the final version of this paper.

### Conflict of interest statement

The authors declare that the research was conducted in the absence of any commercial or financial relationships that could be construed as a potential conflict of interest.
